# Posttraumatic Stress Symptoms, Quality of Life, and Stress Burden in Caregivers of Patients With Severe Mental Illness: An Underestimated Health Concern

**DOI:** 10.3389/fpsyt.2021.623499

**Published:** 2021-04-01

**Authors:** Ahmed Rady, Tarek Mouloukheya, Eman Gamal

**Affiliations:** ^1^Department of Psychiatry, Alexandria University School of Medicine, Alexandria, Egypt; ^2^El Mamoura Psychiatric Hospital, Alexandria, Egypt

**Keywords:** posttraumatic stress disorder, zarit burden interview, quality of life, posttraumatic symptoms diagnostic scale, severe mental illness, care giver

## Abstract

Caregivers of patients with severe mental disorders experience a heavy stress burden that can manifest as psychiatric symptoms mimicking posttraumatic stress disorder (PTSD) and can negatively impact interpersonal relationships and work performance. The present study investigated the prevalence of PTSD symptoms, quality of life (QoL), and stress burden in caregivers of patients with severe mental illness. A total of 70 caregivers of severely mentally ill patients and 70 control subjects who were caregivers of patients with a chronic debilitating medical illness (cardiovascular disease) were recruited from university hospital outpatient facilities. Severe mental illness was defined based on a Global Assessment of Functioning score <50 and duration of illness >2 years. Both groups were evaluated with the Zarit Burden interview, a QoL questionnaire, and Posttraumatic Diagnostic Scale (PDS). The results showed that 37.14% (*n* = 26) of caregivers of patients with severe mental illness showed PTS symptoms compared to 0% of caregivers of patients with physical illness, and 15.17% (*n* = 11) met the diagnostic criteria for PTSD. Caregivers of patients with severe mental illness had higher stress burden and lower QoL scores than the control group (*p* < 0.05). These results indicate that caregivers of patients with severe mental illness have a high stress burden that may lead to PTSD, highlighting the importance of providing psychological support to this group.

## Introduction

Looking after patients with psychiatric disorders is often associated with a lot of sacrifices, as care givers need to adapt their personal lives to care for their patient on detriment of social, family, leisure, and even professional life. Those adaptive coping strategies often remain ungratified and underestimated, which potentially generate diverse psychopathologies on depressive and anxiety dimensions (1).

In a recently published systemic review that included 35 studies to assess psychological impact on caregivers of patients with mental illness, authors highlighted the magnitude of psychological burden found among caregivers of patients with psychiatric disorders, and shed light on the importance of helping those caregivers to develop their sense of coherence as an effective coping mechanism to overcome stress-related responses (2).

The stress burden among caregivers of patients with severe mental illness has been associated with the development of Posttraumatic stress disorder (PTSD) among those caregivers. This observation highlights the magnitude of the burden and its devastating sequelae among caregivers (3–5). Carmassi et al. (6) recently published an interesting systemic review article where Posttraumatic stress disorder (PTSD) in caregivers of patients with severe mental illness was studied and different factors in those caregivers were then subcategorized as modifiable or permanent factors. This new perspective provided a potential research orientation that can help in the development of various psychological interventions to address that issue and prevent PTSD among caregivers of patients with severe mental illness (6).

Posttraumatic stress disorder (PTSD) is a complex and heterogeneous mental illness that often has a poor outcome (7). The lifetime prevalence is estimated as ~4% (8). The psychiatric symptoms of PTSD can be grouped into 3 categories: (1) hypervigilance, which is associated with increased sympathetic tone and constant apprehension, irritability, insomnia, anxiety, and heightened arousal accompanied by a depressive mood; (2) resuscitation or recurrence of memories associated with the trauma, for example in the form of nightmares during sleep or as flashbacks in an awakened state; and (3) avoidance of cues that serve as a reminder or the traumatic event (9).

Various psychological theories have emphasized the importance of priming as a cognitive neurobiologic process that associates a strong sense of insecurity and hopelessness with specific cues from the traumatic event (10). Psychodynamic theories assume that Posttraumatic stress (PTS) represents a failure of unconscious defense mechanisms of the ego to overcome a traumatic event, and the emergence of unhealthy, neurotic defense mechanisms that constitute the core psychopathology of PTSD (11). Data from molecular genetic studies have implicated the dopaminergic, serotonergic, adrenergic, and GABAergic neurotransmission systems in the genetic susceptibility to PTSD (12).

The recent attributes of the traumatizing event, in PTSD, got out of the old restrictions where traumatic event had to be beyond normal experiences and had been replaced by a wider scope where traumatic event was related to the severe subjective feeling of being traumatized with associated helplessness and impending threat weather the event itself was beyond normal experiences or not. In fact, the definition of the traumatizing event that may precipitate PTSD has evolved over the years and now includes a relativity dimension; thus, the event is considered significant as long as it is traumatizing to the patient (13). Traumatic events are typically linked to injuries, war, and natural disasters but is rarely attributed to the experience of providing long-term care for patients with chronic and/or severe mental illness, even though caregivers are frequently subjected to psychological and sometimes physical threats that can arouse feelings of deep distress and helplessness consistent with PTS (6, 14). The imbalance in the relationship between the caregiver and care receiver is aggravated when the latter has cognitive impairment; additionally, the process of caregiving can negatively affect the caregiver's relationships with other family members or partners/spouses (5, 6).

Severe mental illness is defined as a debilitating psychiatric condition associated with severe loss of occupational and interpersonal functioning. Clinical definitions of severe mental illness include a low score on the Global Assessment of Functioning (GAF) scale, often used to assess disability on axis V in DSM 4-R (15), and duration of illness >2 years (16). Severe mental illness is a commonly used term to describe psychopathologies associated with severe disorganization and disruptive behaviors particularly schizophrenia and bipolar disorders. Though the term is not specific to a particular psychopathology, Jackson et al. (17) demonstrated that severe mental illness as a diagnosis based on disability could subtly be detected in words and phrases used in discharge sheets. This finding, in our opinion, confirms indirectly the reliability of parameters such as GAF and duration of illness as objective measures of severe mental illness revealed the resonance between objective and subjective sense of severe mental illness (17). Most families of severely mentally ill patients are unprepared to cope with the onset of the illness. Although caregivers play a key role in reducing the frequency and length of hospitalization as well as self-harm and suicidal behaviors in patients with severe mental illness, caregiving is an exhausting process that can have negative physical, psychological, emotional, social, and financial impacts on caregivers (6, 18). Various strategies may be adopted by caregivers to avoid burnout including educating themselves about the illness and taking care of themselves by maintaining a healthy lifestyle, remaining socially active, accepting help from others, acknowledging their own emotions, engaging in leisure activities away from the caregiving setting (e.g., scheduling a holiday), and seeking assistance from local authorities and social organizations (19).

How different psychiatric disorders are ranked in terms of psychological impact on care givers is still unclear in literature, though some studies are rather focused on a single diagnosis as schizophrenia (20–22), dementia (23–25), or bipolar disorder (21, 26). Recent literature attributed psychological burden among care givers to poor resilience as a psychological construct or inability to demonstrate sufficient compassion to one-self (23, 27). Evidence based practice shows that providing multi-family psychoeducational groups for caregivers of patients with severe mental illness have demonstrated efficacy to alleviate stress burden (28, 29).

Nonetheless, caregivers' efforts are often underestimated and overlooked by society including by the medical community, and is more often viewed as a free healthcare resource (30). As such, there is a lack of information on the mental health burden experienced by caregivers and the resultant public health burden to society.

To address this issue, the present study investigated stress burden, quality of life (QoL), posttraumatic stress (PTS) symptoms, and PTSD diagnosis in caregivers of patients with severe mental illness in Alexandria, Egypt. We have to mention that health facilities, in our country, do not provide caregivers and only those with high financial status can get an assistance with private facilities. Our study has been carried out at the university hospital which is a public free of charge health facility that doesn't provide caregivers to patients who need continuous supervision and accompaniment consecutively, that family members as parents, siblings, or spouses carry out that role.

## Methods

### Subjects

This cross-sectional, comparative, observational study enrolled 70 caregivers of patients with severe mental illness (with a GAF score <50 as it is systematically included in their records), along with 70 caregivers of patients with severe chronic physical illness (Ischemic cardiac disease with ejection fraction (EF) <45% and experiencing symptoms of heart failure as long term sequelae) as a control group. Though cardiac patients enrolled in the study are not particularly touched with stigma issues as the case in mental illness, but they represented a significant burden to care givers in terms of autonomy and need for continuous supervision and accompaniment. Both groups were recruited from outpatient health facilities at Alexandria University hospitals. Caregivers were recruited as they were accompanying their patients to the outpatient health facilities, their role as the main contact caregiver was confirmed from the patients' records. Our sample was homogenous in terms of ethnicity. The subjects were aged 18–65 years. Severe mental illness was defined as a GAF score <50 and duration of illness >2 years. Though World Health Organization Disability Assessment Schedule (WHODAS) used for DSM-5 currently replaced GAF in DSM-4-R but the later is still widely used (31) and provided in patients' records. Demographic data such as age, sex, relationship to the patient, marital status, and employment status were collected. The study was approved by the institutional review board of Alexandria School of Medicine, and was carried out in strict accordance with the Helsinki guidelines. All participants consented in writing to have their data used for research purposes.

### Clinical Assessment

All participants were evaluated with 3 psychometric measurement tools. The Arabic version of the Zarit Burden Interview (ZBI) (32). The ZBI is a commonly used 22-item self-report questionnaire that covers 5 dimensions of stress burden—i.e., psychological, financial, social, health, and relationship with the patient being cared for. The items are scored on 5-point Likert-like scale (33). The Arabic version of the ZBI has been developed with a Cronbach α = 0.77 and concurrent validity with 3 psychological constructs: depression assessed by Hamilton rating scale (HAM-D) (*r* = 0.39, *p* < 0.001), psychological well-being evaluated using the well-being Index (WHO-5) (*r* = 0.37, *p* < 0.001) and emotional burden measured by the exhaustion subscale of Maslach Burnout Inventory (MBI) (*r* = 0.22, *p* < 0.05) (32).

QoL was evaluated using the Physical, Cognitive, Affective, Social, Economic, and Ego/Personality-oriented (PCASEE) QoL self-report questionnaire (34), which consists of 30 items covering 6 dimensions of QoL. It has shown validity to predict relapse, to identify therapeutic objectives and to differentiate among therapies. The Arabic version of the PCASEE QoL was applied (35).

We screened for PTSD symptoms according to DSM-5, with the Arabic version of the self-reported Posttraumatic Diagnostic Scale (PDS) (36), which includes 49 questions in 4 categories: the first 2 assess the traumatic event; the third screens for 3 clusters of PTSD; and the fourth evaluates the degree of functional impairment, with the severity classified as no impairment or mild, moderate, or severe impairment based on the number of (yes/no) answers (0, 1 or 2, 3–6, or 7 or 8 yes answers, respectively). The severity of posttraumatic symptoms was determined based on the total score of the third component of the PDS scale, with scores of <10, 11–20, 21–35, and >36 corresponding to mild, moderate, moderate-to-severe, and severe, respectively (37). The Arabic version of the PDS was developed from the original version published for PDS (37) and it showed acceptable psychometric properties of internal consistency (α = 0.93) and reliability (*r* = 0.81) (36). Patients with severe symptoms in the third component of the PDS scale were evaluated for PTSD with the clinician version of the Structured Clinical Interview for DSM-V Axis I Disorders PTSD module (38).

### Statistical Analysis

Data were analyzed using SPSS v20.0 software (IBM, Armonk, NY, USA). Qualitative data are presented as numbers and percentages, and quantitative data are presented as ranges (minimum and maximum values), mean, standard deviation, and median. The chi-squared test was used to evaluate categoric variables, with Fisher's exact test or the Monte Carlo test used for correction when >20% of cells had an expected count of <5. The Mann–Whitney *U*-test was used for non-normally distributed quantitative (non-parametric) variables. The significance of differences in intergroup comparisons was judged at the 5% level.

## Results

In our sample, 52.9% of patients with severe mental illness were diagnosed as bipolar disorder (*N* = 37) and 47.1% had the diagnosis of schizophrenia (*N* = 33).

### Characteristics of the Study Population

The 2 groups (caregivers of patients with chronic severe mental illness and caregivers of patients with Ischemic cardiac disease complicated by chronic heart failure) didn't show statistical difference for age, duration of illness of patients, sex ratio, marital status, and employment (Table 1).

**Table 1:** Demographic data for caregivers of patients with severe mental illness and patients with cardiac illness (ischemic cardiac disease complicated with chronic heart failure).

	**Caregivers of patients with chronic severe mental illness** ** (*****N*** **=** **70)**		**Caregivers of patients with cardiac illness** ** (*****N*** **=** **70)**		**Test of significance^**†**^**	p^*^
	***N*.**	**%**	***N***.	**%**		
**Sex**						
Male	23	32.9	21	30.0	χ^2^ = 0.133	0.716
Female	47	67.1	49	70.0		
**Marital status**						
Single	1	1.4	2	2.9	χ^2^ = 2.25	p_MC_ = 0.611
Married	61	87.1	58	82.9		
Divorced	1	1.4	4	5.7		
Widowed	7	10.0	6	8.6		
**Level of education**						
Illiterate	21	30.0	13	18.6	χ^2^ = 2.54	0.281
					χ^2^ = 104.8^*^	*P* < 001
School	29	41.4	35	50.0		
High school	20	28.6	22	31.4		
**Caregiver relation**						
Mother	27	38.6	0	0.0	χ^2^ = 104.802^*^	<0.001^*^
Father	9	12.9	0	0.0		
Sister	11	15.7	0	0.0		
Brother	11	15.7	0	0.0		
Wife	9	12.9	25	35.7		
Daughter	0	0.0	24	34.3		
Husband	3	4.3	8	11.4		
Son	0	0.0	13	18.6		
**Employment**						
Employed	37	52.9	30	42.9	χ^2^ = 1.4	0.236
Unemployed	33	47.1	40	57.1		
**Duration of illness**	5.76± 2.71		5.51 ± 3.42		*U* = 2,196	0.285
**Age, years**	46.06 ±9.55		43.37 ±9.28		*t* = 1.687	0.094

† *Both arms were balanced in terms of age, duration of illness of patients, sex ratio, marital status, and employment. χ^2^, chi-squared test; MC, Monte Carlo; t, Student's t test; U, Mann–Whitney test*.

* *Statistically significant at p ≤ 0.05*.

### Stress Burden in Both Care Givers Groups

Comparing stress burden in care givers showed the total score of the Zarit scale to be 46.54 ± 18.27 in care givers of patients with severe mental illness and 28.94 ± 13.57 in care givers of cardiac patients. The burden was significantly higher among care givers of patients with severe mental illness (*U* = 1098; *P* < 001) (Table 2).

**Table 2:** Stress burden in caregivers of patients with severe mental illness and patients with cardiac illness (ischemic cardiac disease complicated with chronic heart failure).

**Zarit scale**	**Caregivers of patients with chronic severe mental illness (*N* = 70)**	**Caregivers of patients with cardiac illness** ** (*N* = 70)**	**Test of significance^**†**^**	p^*^
Total score (M ± SD)	46.54 ± 18.27	28.94 ± 13.57	*U* = 1,098^*^	<0.001
**Distribution according to severity**				
None (0–20)	10	29	χ^2^ = 33.9^*^	<0.001
Mild (21–40)	17	31		
Moderate (41–60)	27	7		
Severe (61–88)	16	3		

† *χ^2^, chi-squared test; U, Mann–Whitney test*.

* *Statistically significant at p ≤ 0.05*.

### Quality of Life Assessment in Both Care Givers Groups

Self-reported quality of life among care givers with PCASEE quality of life scale found a total score of 87.96 ± 25.60 and 114.50 ± 20.94 in care givers of patients with severe mental illness and cardiac patients, respectively. A significantly more poor quality of life was found among care givers of patients with severe mental illness (*U* = 1,054; *P* < 001). This significant difference was found across the six subdomains of the PCASEE scale (Table 3).

**Table 3:** Self-reported quality of life of caregivers of patients with severe mental illness and cardiac illness (ischemic cardiac disease complicated with chronic heart failure).

**PCASEE quality of life scale subscales**	**Caregivers of patients with severe mental illness** ** (*N* = 70)**	**Caregivers of patients with cardiac illness** ** (*N* = 70)**	***U*^**†**^**	p^*^
Self problems	14.47 ± 4.03	19.07 ± 3.84	966.00^*^	<0.001
Economic problems	14.46 ± 6.09	19.19 ± 5.25	1358.50^*^	<0.001
Social problems	14.46 ± 5.38	18.66 ± 4.42	1277.00^*^	<0.001
Emotional problems.	13.57 ± 5.14	18.30 ± 3.58	1161.00^*^	<0.001
Cognitive problems	15.06 ± 5.32	18.89 ± 4.44	1443.50^*^	<0.001
Somatic problems	15.94 ± 5.05	20.40 ± 3.75	1199.00^*^	<0.001
Total score	87.96 ± 25.60	114.50 ± 20.94	1054.0^*^	<0.001

*Values are shown as mean ± standard deviation*.

† *Mann–Whitney test*.

* *Statistically significant at p ≤ 0.05*.

*PCASEE, Physical, Cognitive, Affective, Social, Economic, and Ego/Personality-oriented*.

### PTSD, Stress Burden, and QoL in Caregivers of Severely Mentally Ill Patients

According to PDS scores, 15.17% (*n* = 11) of caregivers of patients with severe mental illness met the diagnostic criteria for PTSD compared to 0% of those caregivers of patients with cadric disease. Among caregivers of patients with severe mental illness who met the diagnostic criteria of PTSD, 2 had moderate and 9 had moderate-to-severe symptoms; 1 had mild functional impairment while 8 were moderately impaired and 2 were severely impaired. Prevalence of PTSD was significantly higher among care givers of patients with severe mental illness compared to the control group (χ^2^ = 11.93; *P* < 001) (Table 4).

**Table 4:** Posttraumatic diagnostic scale (PDS) in both care givers of patients with cardiac illness (ischemic cardiac disease complicated with chronic heart failure).

	**Care givers of patients with severe mental illness (*****N*** **=** **70)**		**Care givers of patients with severe cardiac illness** ** (*****N*** **=** **70)**		**Test of sig**.	***p***
	***N***.	**%**	***N***.	**%**		
**PTSD found**	11	15.7	0	0.0	χ^2^ = 11.93^*^	<0.001^*^
**PTSD not found**	59	84.3	70	100		
**Care givers of patients with severe mental illness who met diagnosis of PTSD (*****N*** **=** **11)**						
**PTSD severity**						
Mild	0	0.0				
Moderate	2	18.2				
Severe	0	0.0				
Mod - severe	9	81.8				
**Degree of impairment**						
Mild	1	9.1				
Moderate	8	72.7				
Severe	2	18.2				

*χ^2^ Chi square test*;

* *Statistically significant at p ≤ 0.05*.

### Comparison Between Caregivers Who Had PTSD With Those Who Did Not Meet Diagnostic Criteria for PTSD, Among Caregivers of Patients With Sever Mental Illness

Zarit total score was 56.82 ± 10.63 in care givers who met diagnosis of PTSD compared to 44.63 ± 18.81 in patients who didn't have PTSD. The former group had a significantly higher stress burden (*U* = 149.5; *P* < 0.05). As for duration of illness it was 4.0 ± 2.45 and 6.08 ± 2.65 years in care givers who had and those who had not PTSD, respectively. The difference was significant (*U* = 154; *P* < 0.05) (Table 5).

**Table 5:** Duration of illness and Zarit Burden in caregivers of patients with severe mental illness with or without a PTSD diagnosis.

	**Care givers of patiens with severe mental illness (*****N*** **=** **70)**		***U*^**†**^**	***p***
	**Those who didn't have PTSD** ** (*N* = 59)**	**Those who had PTSD** ** (*N* = 11)**		
Duration of illness	6.08 ± 2.65	4.0 ± 2.45	154.0^*^	0.005^*^
Zarit Burden	44.63 ± 18.81	56.82 ± 10.63	149.5^*^	0.036^*^

*Values are shown as mean ± standard deviation*.

† *Mann–Whitney test*.

* *Statistically significant at p ≤ 0.05*.

## Discussion

The present study investigated the mental health status of caregivers of patients with severe mental illness, as the psychological burden experienced by this group is rarely addressed. We found that these caregivers had a high stress burden as determined with the ZBI, which is in accordance with results from other studies evaluating illness burden in various ethnic and cultural backgrounds (39, 40). Interestingly, stress burden varies according to the socioeconomic status of the countries in which the study is carried out, with developing countries showing a higher burden than developed countries. This can be attributed to higher education levels, better health facilities, greater availability of social assistance resources, and less stigma surrounding mental illness in the latter (39–41). Stigma as a culture-sensitive issue contributes to high stress burden among caregivers of patients with severe mental. Stigma has been shown to negatively affect patients as well as their first-degree relatives and caregivers (42).

The prevalence of Posttraumatic stress (PTS) symptoms in caregivers of patients with severe mental illness in our study was 37.1% and near half of them (15.7%) met diagnostic criteria of PTSD. A previous study reported PTSD in 52% of care givers of patients with psychosis who have been subject to moderate to severe aggression (43). In a review overlooking 8 studies evaluating PTSD among care givers of patients with bipolar disorder revealed a varying prevalence rate ranging between 7 and 40% (44). Variation in reported prevalence of PTSD, in care givers of patients with mental illness, may be attributable to cultural differences and variable criteria for the diagnosis of psychiatric disorders, whereby a greater traumatizing effect with more severe PTS symptoms may be required for a diagnosis of PTSD in some situations. Posttraumatic symptoms have also been linked to the level of violence in patients with psychotic disorder (45), which is beyond the scope of our study but nonetheless warrants consideration.

In the present work we examined the mental health status of caregivers of patients with severe mental illness, with caregivers of patients with cardiac illness serving as a control group. Such a comparison is scarce in the literature. In the present study, caregivers of patients with cardiac disease were their spouses in 47.1% and their siblings in 52.9%, while among care givers of patients with severe mental illness the majority was parents (51.5%), followed by brothers and sisters (31.4%) and finally, only 17.2% were marital partners (wives or husbands). This difference may contribute to the higher PTSD in care givers of patients with severe mental illness often presenting, for first time, in the 2nd and 3rd decades of life. In a recently published review that included 31 studies with care givers of young adult patients and children showed that younger age among patients was a major risk factor for PTSD in their care givers, and interestingly, younger age of care givers themselves was another risk factor to develop PTSD (6). Such findings support ours, as psychiatric patients included in our study had schizophrenia (47.1%) and bipolar disorder (52.9%), both psychopathologies hit at a young age, compared to ischemic cardiac disease.

We found an association between PTSD and other parameters contributing to stress burden such as illness duration. This is in agreement with findings from studies carried out in other populations (27, 46, 47), indicating that the stress burden experienced by caregivers is independent of cultural factors, though transcultural larger scale multi-center studies are needed to confirm such presumption. The higher stress burden as measured by total scores of the ZBI scale and its subscales and lower QoL scores in caregivers of patients with severe mental illness group suggests differences in the mental representation of illness and the way it is perceived by caregivers. QoL is a multidimensional concept that is measured using different methods. The low QoL scores are in agreement with findings from studies evaluating the psychological and physical impacts of caring for patients with psychosis, among other mental illnesses (48, 49). A low QoL can be attributed to the adaptation that is required of caregivers so that they can carry out their caregiving duties, including self-denial of pleasurable activities and other examples of self-sacrifice that often go unrecognized (48, 49).

Most studies evaluating the well-being of caregivers of patients with psychiatric disorders have focused on patients with dementia where sleep disturbances, behavioral changes, and psychotic episodes were shown to have devastating effects on caregivers (50). However, dementia has it's specificity among other psychiatric illnesses because of the nature of the disorder (neurodegenerative) and the age group that is typically affected (the elderly) (50, 51). Our study is significant because it specifically examined the mental health status of caregivers of patients with chronic and severe mental illness, which is rarely addressed. Moreover, our results underscore the importance of providing psychological support to this group, in light of their high stress burden.

Wang et al. (20) has shown that levels of burden as perceived by caregivers of patients with schizophrenia was correlated with frequency of hospitalization and general poor outcome among their patients (20). This area of research is in need of exploration on a wider scale and on diverse psychopathologies. The relation between burden in caregivers and relapse rates of their patients is not fully studied. In an interesting study that looked at this reciprocal causality from a different perspective found that subjective sense of stress burden among caregivers of rapid cycling bipolar patients was significantly associated with their sense of responsibility because care givers considered themselves morally-responsible of assuring regular intake of medications by their patients (52).

Awareness of the impact of caregivers' burden emphasizes that families and caregivers treatment pathways should become more commonplace and health insurers should include these in services available under their health plan coverage. Future researches on psychological programs for care givers can potentially strengthen their coping strategies through individual, group, or community based interventions. Those caregivers-focused interventions can improve well-being of caregivers and subsequently their ability to better take care of their patients.

Our study has some limitations that needs to be addressed in future studies. The sample was small and didn't allow for generalization of the results. The sample size didn't allow us to explore the relation between different psychometric data as stress burden, PTS symptoms… with specific subcategories as age segments, genders and sociodemographic groups, different psychiatric disorders. Division of our sample into subcategories leads to smaller comparative groups which weakens the statistical analysis. Our study didn't include some important confounding factors such as violence rates among patients as well as stigmatization and resilience among caregivers. Another limitation is the cross sectional design which doesn't evaluate the longitudinal relation between patients with severe mental illness and psychological burden among caregivers. Our study relied on self-reported questionnaire that could be strengthened by objective physician-reported scales.

## Data Availability Statement

The original contributions presented in the study are included in the article/Supplementary Material, further inquiries can be directed to the corresponding author/s.

## Ethics Statement

The studies involving human participants were reviewed and approved by Review board of Alexandria school of Medicine. The patients/participants provided their written informed consent to participate in this study.

## Author Contributions

AR wrote the manuscript. All authors equally contributed to study design and analysis of data and interpretation of results.

## Conflict of Interest

The authors declare that the research was conducted in the absence of any commercial or financial relationships that could be construed as a potential conflict of interest.
